# 1-{[(Cyclo­hexyl­oxy)carbon­yl]­oxy}ethyl 3-{[2′-(2-ethyl-2*H*-tetra­zol-5-yl)biphenyl-4-yl]meth­yl}-2-oxo-2,3-dihydro-1*H*-benzimidazole-4-carboxyl­ate

**DOI:** 10.1107/S1600536810011049

**Published:** 2010-03-31

**Authors:** A. Mohan, P. Ramesh, D. Saravanan, M. N. Ponnuswamy

**Affiliations:** a Department of Chemistry, National College, Tiruchirappali 620 001, India; b Centre of Advanced Study in Crystallography and Biophysics, University of Madras, Guindy Campus, Chennai 600 025, India

## Abstract

In the title compound, C_33_H_34_N_6_O_6_, the dihydro­benzimidazol-2-one ring system is essentially planar (r.m.s. deviation = 0.021 Å). The cyclo­hexane ring adopts a chair conformation. In the 5-(biphenyl-2-yl)-2*H*-tetra­zole fragment, the tetra­zole ring is twisted away from the attached benzene ring by 35.73 (11)° and the two benzene rings form a dihedral angle of 68.00 (9)°. An intra­molecular C—H⋯O inter­action is observed. In the crystal, the mol­ecules are linked into a zigzag chain running along the *b* axis by inter­molecular N—H⋯O hydrogen bonds.

## Related literature

For applications of tetra­zole derivatives in coordination chemistry, medicinal chemistry and materials science, see: Dunica *et al.* (1991 ▶); Wittenberger & Donner (1993 ▶); Xiong *et al.* (2002 ▶); Xue *et al.* (2002 ▶). For metal-organic coordination compounds with tetra­zole ligands, see: Hu *et al.* (2007 ▶); Lü (2008 ▶). For puckering parameters, see: Cremer & Pople (1975 ▶).
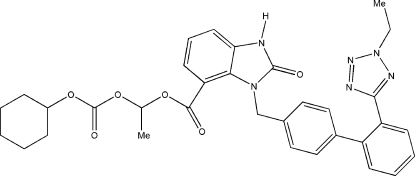


## Experimental

### 

#### Crystal data

C_33_H_34_N_6_O_6_
*M* *_r_* = 610.66
Monoclinic, 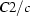

*a* = 16.3770 (7) Å
*b* = 8.5928 (4) Å
*c* = 43.7733 (19) Å
β = 91.150 (1)°
*V* = 6158.7 (5) Å^3^
*Z* = 8
Mo *K*α radiation
μ = 0.09 mm^−1^
*T* = 293 K
0.19 × 0.14 × 0.08 mm

#### Data collection

Bruker Kappa APEXII area-detector diffractometer
Absorption correction: multi-scan (*SADABS*; Sheldrick, 2001 ▶) *T*_min_ = 0.984, *T*_max_ = 0.993
34307 measured reflections
7255 independent reflections
5559 reflections with *I* > 2σ(*I*)
*R*_int_ = 0.026

#### Refinement

*R*[*F* ^2^ > 2σ(*F* ^2^)] = 0.054
*wR*(*F* ^2^) = 0.143
*S* = 0.99
7255 reflections
412 parameters
H atoms treated by a mixture of independent and constrained refinement
Δρ_max_ = 0.29 e Å^−3^
Δρ_min_ = −0.17 e Å^−3^

### 

Data collection: *APEX2* (Bruker, 2004 ▶); cell refinement: *SAINT* (Bruker, 2004 ▶); data reduction: *SAINT*; program(s) used to solve structure: *SHELXS97* (Sheldrick, 2008 ▶); program(s) used to refine structure: *SHELXL97* (Sheldrick, 2008 ▶); molecular graphics: *ORTEP-3* (Farrugia, 1997 ▶); software used to prepare material for publication: *SHELXL97* and *PLATON* (Spek, 2009 ▶).

## Supplementary Material

Crystal structure: contains datablocks global, I. DOI: 10.1107/S1600536810011049/ci5048sup1.cif

Structure factors: contains datablocks I. DOI: 10.1107/S1600536810011049/ci5048Isup2.hkl

Additional supplementary materials:  crystallographic information; 3D view; checkCIF report

## Figures and Tables

**Table 1:** Hydrogen-bond geometry (Å, °)

*D*—H⋯*A*	*D*—H	H⋯*A*	*D*⋯*A*	*D*—H⋯*A*
N16—H16⋯O6^i^	0.85 (2)	2.04 (2)	2.8508 (19)	161 (2)
C20—H20*A*⋯O5	0.97	2.22	3.004 (2)	137

Symmetry code: (i) 
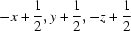
.

**Fig. 1.**
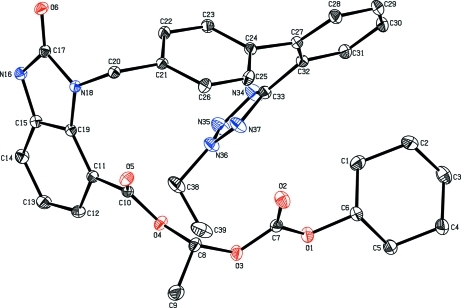
The molecular structure of the title compound, showing displacement ellipsoids at the 30% probability level. H atoms have been omitted for clarity.

**Fig. 2.**
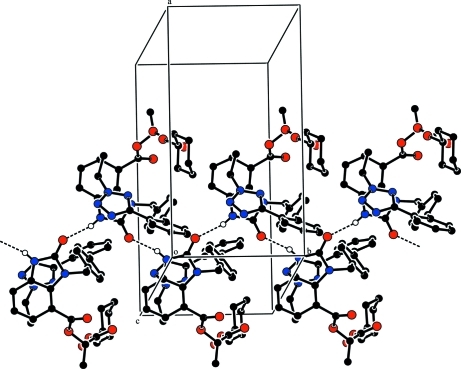
The crystal packing of the title compound, viewed down the c axis. H atoms not involved in hydrogen bonding (dashed lines) have been omitted for clarity.

## supplementary crystallographic information

### Comment

Tetrazole derivatives have attracted considerable attention due to their biological activities. The synthesis of new ligands in this family is an important task for the development of modern coordination chemistry (Hu *et al.*, 2007; Lü, 2008). These compounds also possess wide range of applications in coordination chemistry, medicinal chemistry and materials science (Xiong *et al.*, 2002; Xue *et al.*, 2002; Dunica *et al.*, 1991; Wittenberger *et al.*, 1993). In view of these importance and to ascertain the molecular conformation, an X-ray crystallographic study of the title compound has been carried out.

In the title molecule (Fig.1), the dihydrobenzimidazol-2-one ring system and tetrazole ring are planar. The cyclohexane ring adopts a chair conformation; the puckering parameters (Cremer & Pople, 1975) are: q_2_ = 0.006 (3) Å, q_3_ = -0.564 (3) Å and φ_2_ = 106.1 (1)°. The sum of the bond angles around atoms N16 (358.7°) and N18 (358.0°) of the benzimidazole ring system are in accordance with sp^2^ hybridization. In the 5-(2-biphenyl)-2H-tetrazole fragment, the tetrazole ring is twisted away from the attached benzene ring by 35.73 (11)°, and the two benzene rings form a dihedral angle of 68.00 (9)°. An intramolecular C—H···O interaction is observed.

Atom N16 of the molecule at (x, y, z) donates a proton to atom O6 of the molecule at (1/2-x, 1/2+y, 1/2-z ) forming an intermolecular N—H···O bond which link the molecules into a zigzag chain running along *b* axis as shown in Fig 2.

### Experimental

To a suspension of 1-{[(cyclohexyloxy)carbonyl]oxy}ethyl-2-oxo-1-{[2'-(1H- tetrazol-5-yl) biphenyl-4-yl]methyl}-1*H*-benzimidazole-7-carboxylate (40.0 g) in *N*, *N*-dimethyl formamide (250 ml), potassium carbonate (19 g) and ethyl iodide (16.0 g) were added. The mixture was heated and stirred for 2.5 h at 343–355 K. Then the solid was filtered and washed with cold water. The above solid material (20.0 g) was separated and purified by conventional column chromatography using hexane-ethyl acetate (2:1) as eluent. Single crystals were obtained by recrystallizing the crude product from ethanol by slow evaporation technique.

### Refinement

The N-bound H atom was located in a difference map and refined freely. C-bound H atoms were positioned geometrically (C-H = 0.93-0.98 Å) and allowed to ride on their parent atoms, with U_iso_(H) = 1.5U_eq_(C) for methyl H and 1.2 U_eq_(C) for other H atoms.

### Crystal data

**Table:**

C_33_H_34_N_6_O_6_	*F*(000) = 2576
*M**_r_* = 610.66	*D*_x_ = 1.317 Mg m^−^^3^
Monoclinic, *C*2/*c*	Mo *K*α radiation, λ = 0.71073 Å
Hall symbol: -C 2yc	Cell parameters from 3564 reflections
*a* = 16.3770 (7) Å	θ = 0.9–25.0°
*b* = 8.5928 (4) Å	µ = 0.09 mm^−^^1^
*c* = 43.7733 (19) Å	*T* = 293 K
β = 91.150 (1)°	Plate, colourless
*V* = 6158.7 (5) Å^3^	0.19 × 0.14 × 0.08 mm
*Z* = 8	

### Data collection

**Table:**

Bruker Kappa APEXII area-detector diffractometer	7255 independent reflections
Radiation source: fine-focus sealed tube	5559 reflections with *I* > 2σ(*I*)
graphite	*R*_int_ = 0.026
ω scans	θ_max_ = 28.0°, θ_min_ = 1.9°
Absorption correction: multi-scan (*SADABS*; Sheldrick, 2001)	*h* = −21→20
*T*_min_ = 0.984, *T*_max_ = 0.993	*k* = −11→11
34307 measured reflections	*l* = −56→57

### Refinement

**Table:**

Refinement on *F*^2^	Primary atom site location: structure-invariant direct methods
Least-squares matrix: full	Secondary atom site location: difference Fourier map
*R*[*F*^2^ > 2σ(*F*^2^)] = 0.054	Hydrogen site location: inferred from neighbouring sites
*wR*(*F*^2^) = 0.143	H atoms treated by a mixture of independent and constrained refinement
*S* = 0.99	*w* = 1/[σ^2^(*F*_o_^2^) + (0.0675*P*)^2^ + 3.4615*P*] where *P* = (*F*_o_^2^ + 2*F*_c_^2^)/3
7255 reflections	(Δ/σ)_max_ = 0.017
412 parameters	Δρ_max_ = 0.29 e Å^−^^3^
0 restraints	Δρ_min_ = −0.17 e Å^−^^3^

### Special details

**Table:**

Geometry. All esds (except the esd in the dihedral angle between two l.s. planes) are estimated using the full covariance matrix. The cell esds are taken into account individually in the estimation of esds in distances, angles and torsion angles; correlations between esds in cell parameters are only used when they are defined by crystal symmetry. An approximate (isotropic) treatment of cell esds is used for estimating esds involving l.s. planes.
Refinement. Refinement of *F*^2^ against ALL reflections. The weighted *R*-factor wR and goodness of fit *S* are based on *F*^2^, conventional *R*-factors *R* are based on *F*, with *F* set to zero for negative *F*^2^. The threshold expression of *F*^2^ > σ(*F*^2^) is used only for calculating *R*-factors(gt) etc. and is not relevant to the choice of reflections for refinement. *R*-factors based on *F*^2^ are statistically about twice as large as those based on *F*, and *R*- factors based on ALL data will be even larger.

### Fractional atomic coordinates and isotropic or equivalent isotropic displacement parameters (Å^2^)

**Table:**

	*x*	*y*	*z*	*U*_iso_*/*U*_eq_	
O1	−0.10538 (9)	0.40762 (15)	0.08077 (3)	0.0628 (3)	
O2	−0.08267 (10)	0.24202 (17)	0.11988 (3)	0.0791 (4)	
O3	−0.14741 (9)	0.47031 (16)	0.12506 (3)	0.0666 (4)	
O4	−0.09830 (8)	0.58427 (14)	0.16718 (3)	0.0574 (3)	
O5	−0.05644 (7)	0.45285 (14)	0.20880 (3)	0.0568 (3)	
O6	0.24689 (8)	0.63668 (14)	0.25144 (3)	0.0567 (3)	
C1	0.01658 (15)	0.3068 (4)	0.05704 (6)	0.1087 (10)	
H1A	0.0403	0.2760	0.0766	0.130*	
H1B	0.0329	0.4132	0.0530	0.130*	
C2	0.04851 (15)	0.2007 (5)	0.03206 (7)	0.1128 (11)	
H2A	0.1073	0.2125	0.0308	0.135*	
H2B	0.0371	0.0932	0.0372	0.135*	
C3	0.00998 (16)	0.2383 (3)	0.00197 (6)	0.0852 (7)	
H3A	0.0286	0.1648	−0.0132	0.102*	
H3B	0.0266	0.3417	−0.0043	0.102*	
C4	−0.08144 (16)	0.2316 (3)	0.00346 (5)	0.0871 (7)	
H4A	−0.0982	0.1255	0.0075	0.105*	
H4B	−0.1050	0.2623	−0.0161	0.105*	
C5	−0.11349 (14)	0.3378 (3)	0.02823 (5)	0.0725 (6)	
H5A	−0.1020	0.4453	0.0231	0.087*	
H5B	−0.1722	0.3258	0.0296	0.087*	
C6	−0.07367 (11)	0.2981 (2)	0.05837 (4)	0.0570 (4)	
H6	−0.0895	0.1923	0.0641	0.068*	
C7	−0.10780 (11)	0.3606 (2)	0.10939 (4)	0.0528 (4)	
C8	−0.14875 (11)	0.4568 (2)	0.15726 (4)	0.0564 (4)	
H8	−0.1261	0.3569	0.1641	0.068*	
C9	−0.23394 (14)	0.4783 (3)	0.16790 (5)	0.0839 (7)	
H9A	−0.2553	0.5749	0.1601	0.126*	
H9B	−0.2340	0.4800	0.1898	0.126*	
H9C	−0.2674	0.3939	0.1606	0.126*	
C10	−0.05646 (9)	0.56936 (19)	0.19372 (3)	0.0443 (3)	
C11	−0.01429 (9)	0.71732 (18)	0.20158 (3)	0.0444 (3)	
C12	−0.05366 (11)	0.8580 (2)	0.19443 (4)	0.0552 (4)	
H12	−0.1034	0.8546	0.1838	0.066*	
C13	−0.02180 (12)	1.0009 (2)	0.20246 (5)	0.0621 (5)	
H13	−0.0505	1.0911	0.1975	0.074*	
C14	0.05270 (12)	1.0113 (2)	0.21790 (4)	0.0572 (4)	
H14	0.0748	1.1072	0.2234	0.069*	
C15	0.09259 (10)	0.87500 (18)	0.22476 (4)	0.0452 (4)	
N16	0.16694 (9)	0.85162 (16)	0.23946 (3)	0.0479 (3)	
H16	0.1995 (13)	0.923 (2)	0.2449 (5)	0.064 (6)*	
C17	0.18479 (10)	0.69756 (18)	0.24083 (3)	0.0444 (3)	
N18	0.11883 (8)	0.62020 (14)	0.22739 (3)	0.0408 (3)	
C19	0.06123 (9)	0.72809 (17)	0.21703 (3)	0.0401 (3)	
C20	0.12542 (10)	0.45543 (17)	0.21919 (3)	0.0429 (3)	
H20A	0.0729	0.4053	0.2217	0.052*	
H20B	0.1646	0.4051	0.2328	0.052*	
C21	0.15208 (9)	0.43559 (17)	0.18655 (4)	0.0424 (3)	
C22	0.22015 (11)	0.5131 (2)	0.17578 (4)	0.0556 (4)	
H22	0.2508	0.5756	0.1890	0.067*	
C23	0.24300 (11)	0.4989 (2)	0.14583 (4)	0.0569 (4)	
H23	0.2884	0.5532	0.1391	0.068*	
C24	0.19952 (10)	0.40521 (19)	0.12553 (4)	0.0484 (4)	
C25	0.13343 (12)	0.3242 (2)	0.13648 (4)	0.0572 (4)	
H25	0.1046	0.2574	0.1235	0.069*	
C26	0.10931 (11)	0.3405 (2)	0.16638 (4)	0.0538 (4)	
H26	0.0637	0.2868	0.1730	0.065*	
C27	0.22306 (10)	0.3941 (2)	0.09282 (4)	0.0502 (4)	
C28	0.25473 (13)	0.2562 (2)	0.08167 (5)	0.0660 (5)	
H28	0.2580	0.1695	0.0944	0.079*	
C29	0.28138 (14)	0.2456 (3)	0.05206 (5)	0.0750 (6)	
H29	0.3026	0.1522	0.0450	0.090*	
C30	0.27686 (13)	0.3714 (3)	0.03306 (5)	0.0713 (6)	
H30	0.2963	0.3645	0.0133	0.086*	
C31	0.24339 (12)	0.5087 (2)	0.04329 (4)	0.0619 (5)	
H31	0.2391	0.5936	0.0302	0.074*	
C32	0.21603 (10)	0.5215 (2)	0.07304 (4)	0.0503 (4)	
C33	0.17652 (10)	0.6676 (2)	0.08233 (4)	0.0518 (4)	
N34	0.19874 (13)	0.8085 (2)	0.07278 (5)	0.0880 (6)	
N35	0.14788 (13)	0.9081 (2)	0.08502 (6)	0.0919 (6)	
N36	0.09842 (10)	0.82671 (19)	0.10133 (4)	0.0671 (4)	
N37	0.11305 (11)	0.67617 (19)	0.10042 (4)	0.0722 (5)	
C38	0.03020 (16)	0.8941 (3)	0.11829 (7)	0.0923 (8)	
H38A	0.0323	0.8566	0.1392	0.111*	
H38B	0.0356	1.0065	0.1187	0.111*	
C39	−0.04796 (16)	0.8525 (3)	0.10426 (9)	0.1114 (10)	
H39A	−0.0501	0.8894	0.0836	0.167*	
H39B	−0.0912	0.8992	0.1155	0.167*	
H39C	−0.0541	0.7414	0.1044	0.167*	

### Atomic displacement parameters (Å^2^)

**Table:**

	*U*^11^	*U*^22^	*U*^33^	*U*^12^	*U*^13^	*U*^23^
O1	0.0817 (9)	0.0544 (7)	0.0523 (7)	0.0067 (6)	0.0012 (6)	−0.0039 (6)
O2	0.1041 (11)	0.0636 (9)	0.0700 (9)	0.0226 (8)	0.0118 (8)	0.0078 (7)
O3	0.0888 (9)	0.0646 (8)	0.0459 (7)	0.0217 (7)	−0.0104 (6)	−0.0073 (6)
O4	0.0671 (8)	0.0548 (7)	0.0496 (7)	−0.0036 (6)	−0.0145 (6)	0.0028 (5)
O5	0.0632 (7)	0.0562 (7)	0.0505 (7)	−0.0091 (6)	−0.0077 (5)	0.0100 (6)
O6	0.0564 (7)	0.0507 (7)	0.0624 (7)	0.0044 (5)	−0.0168 (6)	−0.0005 (5)
C1	0.0600 (13)	0.180 (3)	0.0852 (17)	−0.0025 (16)	−0.0065 (12)	−0.0433 (19)
C2	0.0604 (14)	0.180 (3)	0.099 (2)	0.0255 (17)	0.0073 (13)	−0.036 (2)
C3	0.0959 (17)	0.0853 (16)	0.0755 (14)	0.0040 (13)	0.0323 (13)	−0.0007 (12)
C4	0.0925 (17)	0.1098 (19)	0.0588 (12)	0.0266 (14)	−0.0034 (11)	−0.0175 (12)
C5	0.0751 (13)	0.0808 (14)	0.0617 (12)	0.0164 (11)	0.0018 (10)	−0.0073 (10)
C6	0.0629 (11)	0.0526 (10)	0.0558 (10)	0.0027 (8)	0.0068 (8)	−0.0049 (8)
C7	0.0524 (9)	0.0496 (9)	0.0561 (10)	−0.0003 (8)	−0.0036 (8)	−0.0041 (8)
C8	0.0665 (11)	0.0564 (10)	0.0459 (9)	−0.0010 (8)	−0.0097 (8)	−0.0012 (8)
C9	0.0662 (13)	0.1121 (19)	0.0734 (14)	−0.0081 (13)	0.0012 (11)	−0.0025 (13)
C10	0.0421 (8)	0.0518 (9)	0.0390 (8)	0.0031 (7)	0.0011 (6)	0.0004 (7)
C11	0.0456 (8)	0.0462 (8)	0.0414 (8)	0.0030 (7)	0.0014 (6)	0.0023 (6)
C12	0.0525 (9)	0.0562 (10)	0.0565 (10)	0.0106 (8)	−0.0068 (8)	0.0040 (8)
C13	0.0672 (11)	0.0462 (9)	0.0726 (12)	0.0147 (8)	−0.0048 (9)	0.0080 (9)
C14	0.0693 (11)	0.0377 (8)	0.0644 (11)	0.0019 (8)	−0.0012 (9)	0.0035 (7)
C15	0.0501 (9)	0.0419 (8)	0.0438 (8)	−0.0007 (7)	0.0024 (7)	0.0034 (6)
N16	0.0503 (8)	0.0408 (7)	0.0525 (8)	−0.0040 (6)	−0.0022 (6)	−0.0014 (6)
C17	0.0500 (9)	0.0433 (8)	0.0399 (8)	0.0002 (7)	−0.0016 (6)	0.0012 (6)
N18	0.0466 (7)	0.0368 (6)	0.0390 (6)	0.0014 (5)	−0.0020 (5)	0.0026 (5)
C19	0.0457 (8)	0.0378 (7)	0.0368 (7)	0.0024 (6)	0.0033 (6)	0.0038 (6)
C20	0.0504 (8)	0.0340 (7)	0.0442 (8)	0.0024 (6)	−0.0052 (6)	0.0053 (6)
C21	0.0459 (8)	0.0346 (7)	0.0465 (8)	0.0050 (6)	−0.0045 (6)	0.0020 (6)
C22	0.0500 (9)	0.0607 (10)	0.0560 (10)	−0.0081 (8)	0.0003 (7)	−0.0154 (8)
C23	0.0474 (9)	0.0639 (11)	0.0599 (10)	−0.0083 (8)	0.0076 (8)	−0.0100 (9)
C24	0.0525 (9)	0.0432 (8)	0.0494 (9)	0.0071 (7)	−0.0006 (7)	−0.0021 (7)
C25	0.0700 (11)	0.0511 (10)	0.0503 (9)	−0.0139 (8)	−0.0077 (8)	−0.0038 (8)
C26	0.0619 (10)	0.0481 (9)	0.0513 (9)	−0.0131 (8)	−0.0023 (8)	0.0039 (7)
C27	0.0464 (8)	0.0534 (9)	0.0505 (9)	0.0043 (7)	−0.0017 (7)	−0.0078 (7)
C28	0.0738 (13)	0.0592 (11)	0.0649 (12)	0.0169 (10)	−0.0017 (10)	−0.0096 (9)
C29	0.0766 (13)	0.0744 (14)	0.0741 (14)	0.0201 (11)	0.0048 (11)	−0.0262 (11)
C30	0.0681 (12)	0.0905 (16)	0.0557 (11)	0.0045 (11)	0.0111 (9)	−0.0228 (11)
C31	0.0618 (11)	0.0720 (12)	0.0522 (10)	0.0010 (9)	0.0062 (8)	−0.0037 (9)
C32	0.0446 (8)	0.0547 (10)	0.0515 (9)	0.0012 (7)	0.0023 (7)	−0.0065 (7)
C33	0.0516 (9)	0.0529 (9)	0.0508 (9)	0.0010 (8)	0.0027 (7)	−0.0009 (7)
N34	0.0871 (13)	0.0578 (10)	0.1206 (17)	−0.0016 (10)	0.0389 (12)	0.0022 (11)
N35	0.0909 (14)	0.0528 (10)	0.1329 (19)	0.0002 (10)	0.0299 (13)	−0.0023 (11)
N36	0.0632 (9)	0.0525 (9)	0.0861 (12)	0.0072 (8)	0.0118 (8)	−0.0043 (8)
N37	0.0714 (10)	0.0525 (9)	0.0935 (13)	0.0126 (8)	0.0265 (9)	0.0053 (8)
C38	0.0877 (17)	0.0716 (14)	0.118 (2)	0.0227 (13)	0.0237 (15)	−0.0142 (14)
C39	0.0715 (16)	0.0864 (18)	0.177 (3)	0.0111 (14)	0.0266 (18)	−0.0051 (19)

### Geometric parameters (Å, °)

**Table:**

O1—C7	1.318 (2)	N16—C17	1.357 (2)
O1—C6	1.462 (2)	N16—H16	0.85 (2)
O2—C7	1.188 (2)	C17—N18	1.389 (2)
O3—C7	1.341 (2)	N18—C19	1.3919 (19)
O3—C8	1.415 (2)	N18—C20	1.4652 (19)
O4—C10	1.3432 (18)	C20—C21	1.512 (2)
O4—C8	1.434 (2)	C20—H20A	0.97
O5—C10	1.1992 (19)	C20—H20B	0.97
O6—C17	1.2265 (19)	C21—C26	1.383 (2)
C1—C6	1.482 (3)	C21—C22	1.389 (2)
C1—C2	1.524 (4)	C22—C23	1.376 (2)
C1—H1A	0.97	C22—H22	0.93
C1—H1B	0.97	C23—C24	1.385 (2)
C2—C3	1.485 (4)	C23—H23	0.93
C2—H2A	0.97	C24—C25	1.381 (2)
C2—H2B	0.97	C24—C27	1.494 (2)
C3—C4	1.501 (4)	C25—C26	1.382 (3)
C3—H3A	0.97	C25—H25	0.93
C3—H3B	0.97	C26—H26	0.93
C4—C5	1.519 (3)	C27—C28	1.386 (2)
C4—H4A	0.97	C27—C32	1.399 (2)
C4—H4B	0.97	C28—C29	1.379 (3)
C5—C6	1.499 (3)	C28—H28	0.93
C5—H5A	0.97	C29—C30	1.365 (3)
C5—H5B	0.97	C29—H29	0.93
C6—H6	0.98	C30—C31	1.380 (3)
C8—C9	1.491 (3)	C30—H30	0.93
C8—H8	0.98	C31—C32	1.390 (2)
C9—H9A	0.96	C31—H31	0.93
C9—H9B	0.96	C32—C33	1.473 (2)
C9—H9C	0.96	C33—N37	1.321 (2)
C10—C11	1.484 (2)	C33—N34	1.334 (2)
C11—C19	1.401 (2)	N34—N35	1.316 (3)
C11—C12	1.402 (2)	N35—N36	1.296 (3)
C12—C13	1.377 (3)	N36—N37	1.316 (2)
C12—H12	0.93	N36—C38	1.472 (3)
C13—C14	1.386 (3)	C38—C39	1.453 (4)
C13—H13	0.93	C38—H38A	0.97
C14—C15	1.372 (2)	C38—H38B	0.97
C14—H14	0.93	C39—H39A	0.96
C15—N16	1.381 (2)	C39—H39B	0.96
C15—C19	1.402 (2)	C39—H39C	0.96
			
C7—O1—C6	117.26 (14)	C15—N16—H16	124.8 (14)
C7—O3—C8	117.98 (14)	O6—C17—N16	127.52 (15)
C10—O4—C8	118.04 (13)	O6—C17—N18	126.02 (14)
C6—C1—C2	110.8 (2)	N16—C17—N18	106.46 (13)
C6—C1—H1A	109.5	C17—N18—C19	109.60 (12)
C2—C1—H1A	109.5	C17—N18—C20	120.38 (13)
C6—C1—H1B	109.5	C19—N18—C20	128.06 (12)
C2—C1—H1B	109.5	N18—C19—C11	134.37 (14)
H1A—C1—H1B	108.1	N18—C19—C15	106.18 (13)
C3—C2—C1	111.2 (3)	C11—C19—C15	119.45 (14)
C3—C2—H2A	109.4	N18—C20—C21	111.38 (12)
C1—C2—H2A	109.4	N18—C20—H20A	109.4
C3—C2—H2B	109.4	C21—C20—H20A	109.4
C1—C2—H2B	109.4	N18—C20—H20B	109.4
H2A—C2—H2B	108.0	C21—C20—H20B	109.4
C2—C3—C4	111.1 (2)	H20A—C20—H20B	108.0
C2—C3—H3A	109.4	C26—C21—C22	117.78 (15)
C4—C3—H3A	109.4	C26—C21—C20	121.18 (14)
C2—C3—H3B	109.4	C22—C21—C20	121.04 (14)
C4—C3—H3B	109.4	C23—C22—C21	121.10 (16)
H3A—C3—H3B	108.0	C23—C22—H22	119.4
C3—C4—C5	111.6 (2)	C21—C22—H22	119.4
C3—C4—H4A	109.3	C22—C23—C24	121.07 (16)
C5—C4—H4A	109.3	C22—C23—H23	119.5
C3—C4—H4B	109.3	C24—C23—H23	119.5
C5—C4—H4B	109.3	C25—C24—C23	117.84 (16)
H4A—C4—H4B	108.0	C25—C24—C27	121.32 (15)
C6—C5—C4	109.95 (18)	C23—C24—C27	120.84 (16)
C6—C5—H5A	109.7	C24—C25—C26	121.24 (16)
C4—C5—H5A	109.7	C24—C25—H25	119.4
C6—C5—H5B	109.7	C26—C25—H25	119.4
C4—C5—H5B	109.7	C21—C26—C25	120.92 (16)
H5A—C5—H5B	108.2	C21—C26—H26	119.5
O1—C6—C1	111.23 (17)	C25—C26—H26	119.5
O1—C6—C5	106.85 (15)	C28—C27—C32	118.54 (17)
C1—C6—C5	111.75 (19)	C28—C27—C24	119.85 (16)
O1—C6—H6	109.0	C32—C27—C24	121.59 (15)
C1—C6—H6	109.0	C29—C28—C27	121.0 (2)
C5—C6—H6	109.0	C29—C28—H28	119.5
O2—C7—O1	127.88 (17)	C27—C28—H28	119.5
O2—C7—O3	124.95 (17)	C30—C29—C28	120.42 (19)
O1—C7—O3	107.13 (15)	C30—C29—H29	119.8
O3—C8—O4	102.64 (14)	C28—C29—H29	119.8
O3—C8—C9	109.53 (16)	C29—C30—C31	119.77 (19)
O4—C8—C9	110.35 (17)	C29—C30—H30	120.1
O3—C8—H8	111.3	C31—C30—H30	120.1
O4—C8—H8	111.3	C30—C31—C32	120.61 (19)
C9—C8—H8	111.3	C30—C31—H31	119.7
C8—C9—H9A	109.5	C32—C31—H31	119.7
C8—C9—H9B	109.5	C31—C32—C27	119.62 (16)
H9A—C9—H9B	109.5	C31—C32—C33	118.51 (17)
C8—C9—H9C	109.5	C27—C32—C33	121.80 (15)
H9A—C9—H9C	109.5	N37—C33—N34	111.09 (16)
H9B—C9—H9C	109.5	N37—C33—C32	124.63 (16)
O5—C10—O4	123.38 (15)	N34—C33—C32	124.25 (17)
O5—C10—C11	126.35 (14)	N35—N34—C33	106.52 (18)
O4—C10—C11	110.23 (13)	N36—N35—N34	106.30 (17)
C19—C11—C12	116.65 (15)	N35—N36—N37	113.45 (16)
C19—C11—C10	124.81 (14)	N35—N36—C38	123.69 (18)
C12—C11—C10	118.48 (14)	N37—N36—C38	122.82 (18)
C13—C12—C11	122.79 (16)	N36—N37—C33	102.65 (16)
C13—C12—H12	118.6	C39—C38—N36	111.2 (2)
C11—C12—H12	118.6	C39—C38—H38A	109.4
C12—C13—C14	120.48 (16)	N36—C38—H38A	109.4
C12—C13—H13	119.8	C39—C38—H38B	109.4
C14—C13—H13	119.8	N36—C38—H38B	109.4
C15—C14—C13	117.54 (16)	H38A—C38—H38B	108.0
C15—C14—H14	121.2	C38—C39—H39A	109.5
C13—C14—H14	121.2	C38—C39—H39B	109.5
C14—C15—N16	129.65 (15)	H39A—C39—H39B	109.5
C14—C15—C19	123.08 (15)	C38—C39—H39C	109.5
N16—C15—C19	107.27 (13)	H39A—C39—H39C	109.5
C17—N16—C15	110.47 (14)	H39B—C39—H39C	109.5
C17—N16—H16	124.4 (14)		
			
C6—C1—C2—C3	55.9 (4)	N16—C15—C19—N18	−0.28 (17)
C1—C2—C3—C4	−55.3 (4)	C14—C15—C19—C11	0.0 (2)
C2—C3—C4—C5	55.8 (3)	N16—C15—C19—C11	−179.68 (14)
C3—C4—C5—C6	−55.5 (3)	C17—N18—C20—C21	−92.35 (16)
C7—O1—C6—C1	−85.6 (2)	C19—N18—C20—C21	70.00 (19)
C7—O1—C6—C5	152.22 (17)	N18—C20—C21—C26	−128.84 (15)
C2—C1—C6—O1	−176.0 (2)	N18—C20—C21—C22	50.8 (2)
C2—C1—C6—C5	−56.6 (3)	C26—C21—C22—C23	1.7 (3)
C4—C5—C6—O1	178.24 (19)	C20—C21—C22—C23	−177.95 (16)
C4—C5—C6—C1	56.4 (3)	C21—C22—C23—C24	−0.9 (3)
C6—O1—C7—O2	5.0 (3)	C22—C23—C24—C25	−1.2 (3)
C6—O1—C7—O3	−172.79 (15)	C22—C23—C24—C27	178.11 (17)
C8—O3—C7—O2	10.3 (3)	C23—C24—C25—C26	2.5 (3)
C8—O3—C7—O1	−171.84 (15)	C27—C24—C25—C26	−176.77 (16)
C7—O3—C8—O4	110.92 (17)	C22—C21—C26—C25	−0.3 (3)
C7—O3—C8—C9	−131.85 (19)	C20—C21—C26—C25	179.28 (15)
C10—O4—C8—O3	−150.40 (14)	C24—C25—C26—C21	−1.8 (3)
C10—O4—C8—C9	92.95 (19)	C25—C24—C27—C28	−68.9 (2)
C8—O4—C10—O5	3.3 (2)	C23—C24—C27—C28	111.8 (2)
C8—O4—C10—C11	−174.68 (14)	C25—C24—C27—C32	112.8 (2)
O5—C10—C11—C19	35.4 (3)	C23—C24—C27—C32	−66.5 (2)
O4—C10—C11—C19	−146.73 (15)	C32—C27—C28—C29	2.2 (3)
O5—C10—C11—C12	−141.61 (18)	C24—C27—C28—C29	−176.16 (18)
O4—C10—C11—C12	36.3 (2)	C27—C28—C29—C30	−0.2 (3)
C19—C11—C12—C13	−1.3 (3)	C28—C29—C30—C31	−1.7 (3)
C10—C11—C12—C13	175.95 (17)	C29—C30—C31—C32	1.6 (3)
C11—C12—C13—C14	0.9 (3)	C30—C31—C32—C27	0.4 (3)
C12—C13—C14—C15	−0.1 (3)	C30—C31—C32—C33	−176.57 (17)
C13—C14—C15—N16	179.23 (17)	C28—C27—C32—C31	−2.3 (3)
C13—C14—C15—C19	−0.4 (3)	C24—C27—C32—C31	176.01 (16)
C14—C15—N16—C17	−178.38 (18)	C28—C27—C32—C33	174.63 (16)
C19—C15—N16—C17	1.28 (18)	C24—C27—C32—C33	−7.1 (2)
C15—N16—C17—O6	177.55 (16)	C31—C32—C33—N37	141.6 (2)
C15—N16—C17—N18	−1.74 (17)	C27—C32—C33—N37	−35.3 (3)
O6—C17—N18—C19	−177.75 (15)	C31—C32—C33—N34	−36.2 (3)
N16—C17—N18—C19	1.55 (17)	C27—C32—C33—N34	146.8 (2)
O6—C17—N18—C20	−12.4 (2)	N37—C33—N34—N35	−0.3 (3)
N16—C17—N18—C20	166.86 (13)	C32—C33—N34—N35	177.82 (19)
C17—N18—C19—C11	178.49 (16)	C33—N34—N35—N36	0.5 (3)
C20—N18—C19—C11	14.6 (3)	N34—N35—N36—N37	−0.6 (3)
C17—N18—C19—C15	−0.78 (16)	N34—N35—N36—C38	−178.2 (2)
C20—N18—C19—C15	−164.66 (14)	N35—N36—N37—C33	0.5 (3)
C12—C11—C19—N18	−178.42 (16)	C38—N36—N37—C33	178.1 (2)
C10—C11—C19—N18	4.6 (3)	N34—C33—N37—N36	−0.1 (2)
C12—C11—C19—C15	0.8 (2)	C32—C33—N37—N36	−178.19 (17)
C10—C11—C19—C15	−176.23 (14)	N35—N36—C38—C39	109.2 (3)
C14—C15—C19—N18	179.41 (16)	N37—N36—C38—C39	−68.2 (3)

### Hydrogen-bond geometry (Å, °)

**Table:**

*D*—H···*A*	*D*—H	H···*A*	*D*···*A*	*D*—H···*A*
N16—H16···O6^i^	0.85 (2)	2.04 (2)	2.8508 (19)	161 (2)
C20—H20A···O5	0.97	2.22	3.004 (2)	137

Symmetry codes: (i) −*x*+1/2, *y*+1/2, −*z*+1/2.
